# Psychosis After Mild Traumatic Brain Injury and the Role of an Integrated Brain Medicine Clinic

**DOI:** 10.1155/crps/8175418

**Published:** 2025-03-09

**Authors:** Carl Froilan D. Leochico, Adrian I. Espiritu, Sarah E. Levitt, Sabrina Lemire-Rodger, Meiqi Guo, Sara B. Mitchell

**Affiliations:** ^1^Azrieli Brain Medicine Fellowship Program, Department of Psychiatry, Sunnybrook Health Sciences Centre, Toronto, Ontario, Canada; ^2^Department of Rehabilitation Medicine, Philippine General Hospital, University of the Philippines Manila, Manila, Philippines; ^3^Department of Physical Medicine and Rehabilitation, St. Luke's Medical Center, Quezon City, Philippines; ^4^Department of Psychiatry, University of Toronto, Toronto, Ontario, Canada; ^5^Centre for Mental Health, University Health Network, Toronto, Ontario, Canada; ^6^Toronto Rehabilitation Institute, University Health Network, Toronto, Ontario, Canada; ^7^Division of Physical Medicine and Rehabilitation, Department of Medicine, University of Toronto, Toronto, Ontario, Canada; ^8^Neurology Quality and Innovation Lab (NQIL), Division of Neurology, Department of Medicine, University of Toronto, Toronto, Ontario, Canada

**Keywords:** complex brain disorder, interdisciplinary care, outpatient clinic, psychosis, schizophrenia, traumatic brain injury

## Abstract

Psychosis after mild traumatic brain injury (TBI) can be rare, complex, and functionally impairing, often requiring inputs from various specialties. This usually entails separate visits and long wait times. We present the case of an elderly patient with schizophrenia-like psychosis after a mild TBI. Three years after the TBI, the treating physiatrist requested diagnostic clarifications and treatment recommendations from a brain medicine clinic (BMC), a novel integrated virtual clinic composed of neurology, psychiatry, and other brain-related disciplines. Six months later, the patient was overall improved, and her driver's license, which had been suspended 6 months after the TBI, was reinstated. We discuss diagnostic and treatment challenges of TBI. BMCs could provide timely, comprehensive, and efficient access to multispecialty care and resources for patients with complex brain disorders and minimize the artificial siloes in healthcare.

## 1. Introduction

Traumatic brain injury (TBI), the “silent epidemic,” has a global age-standardized prevalence of 759 (95% uncertainty interval 731–788) per 100,000 population [1]. According to the Global Burden of Disease consortium, TBI is responsible for 8.1 million years lived with disability and a disability rate of 111/100,000 [1]. TBI rates are the highest in central and eastern Europe and central Asia, and low- and low–middle-income countries have about three times more total TBIs [1]. Global incidences related to falls, road injuries, and violence among others and associated healthcare costs are increasing [1]. Hence, there is a need to provide timely and efficient use of limited healthcare resources.

Schizophrenia-like psychosis, typically presenting with hallucinations and/or delusions [2, 3], is a rare but generally distressing and functionally impairing sequela post-TBI [2, 4]. As complex brain disorders, including post-TBI psychosis, may have multifactorial etiologies [4], patients often require consultation with different brain-related specialties, depending on the clinical presentation [5]. However, the traditional siloed approach, that is, referral to one specialist at a time, may create several barriers to healthcare, such as long wait times, miscommunications, incoordination, service duplications, poor resource allocation, and fatigue of patient or caregiver [6].

The brain medicine clinic (BMC) at Sunnybrook Health Sciences Centre, Toronto, Canada, is a novel service delivery model that leverages shared healthcare for patients with complex brain disorders by integrating “knowledge across disciplines,” which do not only include neurology and psychiatry but also other specialties that treat the same organ, that is, the brain, like physiatry or physical medicine and rehabilitation, geriatric medicine, and neurosurgery as needed [5]. We define complex brain disorders as having symptoms in two or more of the following domains: affect, behavior, and cognition [5]. To minimize artificial siloes in the traditional discipline-specific healthcare setting, the BMC serves as a pathway for patients to receive real-time, integrated, and holistic consultations. We hereby present a case demonstrating the role of an expanded shared care model for brain-related clinical questions that can otherwise take an unnecessary delay to be answered.

## 2. Case Presentation

We report on a woman in her early 70s, who was previously well, functionally independent, and working as a waitress. Past medical history included osteopenia, mitral regurgitation, cataracts, cervical cancer, and remote head traumas without known neurological injuries. The patient had a history of childhood abuse. She finished middle school at grade 8 level and did not pursue further education as she started working as a house cleaner. There was no prior substance use or psychiatric disorder. The family history included bipolar disorder in a sister but no neurodegenerative diseases. The patient and her common-law partner who separated many years ago had two children, including a son whom she placed for adoption at birth and reconnected with later in life. The son passed away in his 40s from a cardiac arrest of unknown cause a few years before the contact with our clinic, while her daughter has been living separately with her own family.

**Figure 1:**
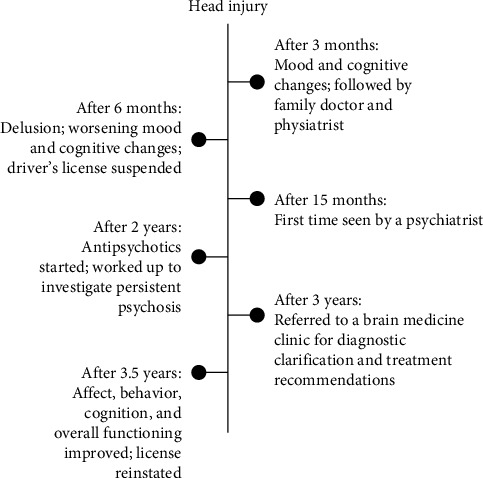
Patient's clinical course.

**Figure 2:**
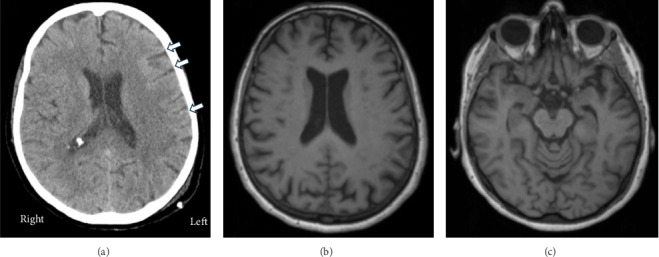
Head computed tomography taken a few hours postinjury shows very subtle subarachnoid hemorrhage on the left frontoparietal sulci (A). T1-weighted magnetic resonance brain imaging taken 2 years postinjury shows mild generalized atrophy more pronounced in the posterior frontal and parietal cortices (B) and the left hippocampus (C).

The patient's clinical course is summarized in Figure 1. Three years prior to referral to the BMC, she suffered a motor vehicular accident. She had no loss of consciousness but had posttraumatic amnesia for <24 h. She scored 14/15 (−1 for confusion) on the Glasgow Coma Scale [7] at the emergency department and was admitted for polytrauma, including midclavicular, rib, pelvic, and acetabular fractures. Head computed tomography showed trace subarachnoid hemorrhage in the left frontoparietal sulci (Figure 2A). The patient had some mobility impairment and cognitive changes including slowed processing and difficulties with multitasking as well as working memory. She was managed conservatively and discharged medically stable from acute care after a week. She then started inpatient followed by outpatient rehabilitation, which improved her mobility.

Three months postinjury, the patient reported photosensitivity and musculoskeletal pains, relieved by acetaminophen as needed. She also endorsed getting easily frustrated and occasionally sad. Her family physician attributed the mood changes to the recent trauma and recommended psychosocial interventions.

Six months postinjury, the patient developed ideas of reference and mild paranoia, with slightly worsening mood and cognitive difficulties. Fifteen months postinjury, she got to see a psychiatrist and was assessed to have moderate depression without psychotic features and mild neurocognitive disorder post-TBI. She was recommended the use of either escitalopram, citalopram, or sertraline which she did not pursue, to avoid tetrahydrocannabinol/cannabidiol (THC/CBD) oil and cream which she had been using postinjury, monitoring of the delusions and paranoia, psychotherapy, social support, and physiatry follow-up.

Around 2 years postinjury, the delusions and paranoia had become more frequent. She had been suspicious of her rehabilitation therapists doing something behind her back and would swear and yell at them. She had not posed any acute psychiatric safety concerns. The patient was started by her psychiatrist on quetiapine 12.5 mg/day, which she discontinued due to sleep and cognitive side effects. She then followed up with her family physician, who prescribed paliperidone 3 mg/day, which only slightly improved her symptoms. The patient's affective, behavioral, and cognitive changes continued and impacted her rehabilitation and daily activities. This made her physiatrist thinking of possible differential diagnoses like frontotemporal dementia, TBI-related psychosis, or THC-/CBD-related psychosis.

In the interim, further investigations were done. Brain magnetic resonance imaging (MRI) demonstrated chronic microangiopathic changes, tiny microhemorrhage in the right corona radiata, and mild generalized atrophy especially in the posterior frontal and parietal lobes (Figure 2B) and left hippocampus (Figure 2C). Blood testing showed hypercholesterolemia and normal levels of vitamin B12 and thyroid hormones.

Three years postinjury, the physiatrist referred the patient to the BMC for diagnostic clarification and treatment recommendations. On initial consult, the patient reported that somebody was listening to her phone calls, that things on the Internet referred to her, that there was theft from her bank account, and that she was being persecuted. She acknowledged that at times she wondered if these experiences were real or not. There were no symptoms of depression, generalized anxiety, panic attacks, manic/hypomanic episodes, hallucinations, aggressions, or suicidal/homicidal ideations. The patient endorsed mild inattention, working memory deficits, and word-retrieval pauses but without any apraxia, visuospatial, or executive function issues. She denied questions assessing sensorimotor, sleep, or autonomic disturbances. She remained independent in basic activities of daily living but required support in some instrumental activities and frequent reassurance from her rehabilitation team due to paranoia. Her driver's license had been suspended due to her persistent psychiatric symptoms.

We saw the patient alert, well-kempt, coherent, and euthymic with reduced affect. Speech had normal rate, rhythm, and volume. She was tangential but redirectable without ongoing hallucinations or delusions. The patient said that she could challenge her intrusive thoughts and consider alternative beliefs during episodes of delusions. Insight and judgment were reasonable. On the Montreal Cognitive Assessment [8], she lost 5 points in the attention domain, 2 points in language, and 1 point in delayed recall. On the Behavioral Neurology Assessment-Short Form [9], she scored 97/110 (executive function, −7; attention, −4; and memory, −2). The rest of the neurological examination was unremarkable.

Based on the integrated assessment of our interdisciplinary team, the patient did have risk factors for post-TBI psychosis including advanced age, complicated TBI, remote concussions, and psychological trauma. Given these risk factors along with the lack of past psychiatric disorders prior to her TBI, we deemed that her presentation was consistent with post-TBI psychosis.

Alternative diagnoses were discussed. Atypical presentation of Alzheimer's disease might be a possibility, especially in the context of hippocampal atrophy on neuroimaging. A vascular component was also brought forward in the setting of an early presentation of disinhibition and difficulties with executive function. Lewy body dementia was also considered in the context of psychosis. Overall, however, a neurodegenerative process was less likely, especially in view of the patient's relative cognitive stability over time. The patient's presentation also did not fit with a primary psychiatric disorder in that she remained to have good insight into her ideas of reference and did not exhibit fixed false beliefs or persistent delusions.

We recommended the following: formal neuropsychological assessment to better understand the patient's cognitive performance in comparison with people of her similar age and educational attainment; repeat brain MRI to determine progressive regional atrophy patterns and for surveillance; brain single-photon emission computed tomography (SPECT) to detect regional hypoperfusion patterns; and electroencephalogram (EEG) to rule out posttraumatic epilepsy given the intermittent nature of her symptoms. Even though we were not considering a primary psychiatric disorder, we recommended increasing her daily paliperidone from 3 to 6 mg/day for symptom management. We also advised a structured cognitive rehabilitation program and lifestyle modifications that could positively impact brain health, such as proper nutrition, adequate sleep, regular physical activity, and social interactions.

On follow-up after 4 months, the patient's affect, behavior, cognition, and overall daily functioning had gradually improved. She still had occasional ideas of reference, but they were more self-redirectable. There were no hallucinations and psychiatric safety concerns. She continued to be well-supported by her local rehabilitation team. Her driver's license was eventually reinstated.

The EEG showed intermittent slowing over the left frontotemporal region with occasional sharply contoured discharges suspicious for, but never frankly, epileptiform. The brain SPECT did not show any blood flow pattern of dementia. A second brain MRI did not show any changes from the previous investigation, demonstrating mild chronic microangiopathic changes, tiny chronic microhemorrhage in the right corona radiata, and mild stable generalized atrophy.

Neuropsychological assessment revealed a cognitive profile characterized by intact memory, visuospatial, and verbal abilities, alongside weaknesses in attention, working memory, processing speed, and inhibitory control. These findings are consistent with sequels of mild-to-moderate TBI, compounded by emotional distress and psychosis-like features. Importantly, there was no evidence of neurodegenerative processes, such as Alzheimer's disease, based on the patient's cognitive strengths, that is, memory and semantic fluency. The patient was eventually discharged from the BMC and continued to be followed by her local psychiatrist and physiatrist.

## 3. Discussion

Based on the Diagnostic and Statistical Manual of Mental Disorders, Fifth Edition (DSM-5), psychotic disorder due to TBI (PDTBI) must include (1) the presence of hallucinations or delusions; (2) evidence from the case history, physical, or laboratory investigations of a direct physiological consequence of TBI; and (3) that the psychosis could not be explained by another mental disorder and did not occur exclusively in delirium [2–4]. Although our patient met these criteria, attributing psychosis directly to TBI is generally often difficult as the relationship between psychosis and TBI can be multifactorial. Considering the biopsychosocial framework, the following factors can be considered: age (younger individuals are at greater risk; mixed results); sex (males at greater risk; mixed results); family history of mental illness; pre-existing psychiatric disorders; prior head traumas; and severity and area(s) of brain injury among others [2, 4, 10]. The diagnosis is often framed as “psychosis associated with, or after, TBI”, that is, posttraumatic or post-TBI psychosis rather than “psychosis due to TBI” [7]. Due to these ambiguities in semantics or diagnostic framing, PDTBI incidence varies widely (0.7%–8.9%) [2]. Furthermore, the available data come from studies limited by retrospective designs, disparities in diagnostic criteria used, small samples, and potential selection bias, that is, including samples with specific characteristics, such as war veterans [11]. Without clear operational definitions, it is indeed challenging to distinguish patients with psychosis attributable to the head injury from those with primary psychosis who have suffered a head injury.

Nonetheless, given our patient's symptom onset and lack of clear premorbid or comorbid psychiatric disorders that could better explain her symptoms, our primary working impression was post-TBI psychosis. She did not have insight impairment, the hallmark of schizophrenia [12]. In addition, schizophrenia typically begins during late adolescence or early adulthood, but the onset may also be late or very late, that is, after the age of 44 or 65 years, respectively. Psychosis onset at these ages is usually secondary to general medical conditions like dementia. A neurodegenerative process, however, was considered unlikely at this time given our patient's cognitive and functional stability. However, she needs to be followed given her risks for future dementia in the context of her advancing age, low education, early-life traumas, head injuries, and hippocampal atrophy.

Generally, mild cases of TBI occur in more than two-thirds of reported cases, with the rest divided equally between moderate and severe ones [13]. While it is recognized that the risk for developing post-TBI psychosis is directly related to the severity of head injury, that is, psychosis onset being more likely in severe TBI [14], schizophrenia is rarely seen following mild TBI. Mild TBI can be diagnosed with negative neuroimaging; however, when a head computed tomography or MRI is completed and demonstrates trauma-related findings, patients are diagnosed with mild TBI “with neuroimaging evidence of structural intracranial injury” (previously known as “complicated” mild TBI) [15].

On literature review, we found that among 69 published cases with post-TBI psychosis, the majority had brain MRI findings of focal lesions or atrophy especially within the frontal or temporal lobes [3]. In addition, about 70% of them had EEG findings similar to our case, that is, focal temporal or frontal slowing, and nearly 30% developed seizures [3]. The hippocampus is particularly vulnerable in TBI because of mechanical forces and other injury-related complications such as increased intracranial pressure, hypoxia, and secondary injury cascades [16]. Disruptions of brain networks, for example, prefrontal and frontal systems, temporal lobes, basal ganglia, mesocortical and mesolimbic systems, and the neurotransmission pathways, projected in these areas cannot only result in cognitive and depressive disorders but also behavioral and psychotic symptoms [11, 16]. All patients post-TBI would be virtually susceptible, but those with a genetic predisposition would have a lower threshold for symptom emergence [11].

In terms of the temporal relationship between TBI and the onset of psychosis, symptoms occurring in the acute phase are likely related to delirium. On the other hand, more than 50% of the patients reported in a case series had symptom onset in the chronic phase within the first year of trauma. Meanwhile, other studies have found latency periods lasting beyond 50 months or even several decades postinjury [11]. Patients typically present with positive symptoms, where delusions are the most frequent (most often persecutory, followed by reference, control, and grandiosity), hallucinations (auditory being more common than visual), and aggressive behaviors [11, 14]. Negative symptoms, for example, blunted affect, avolition, apathy, anhedonia, and asociality, are uncommon [14].

Post-TBI psychosis can be successfully treated, but a thorough diagnostic evaluation is crucial to ascertain its underlying cause and determine the most appropriate, patient-tailored therapeutic approach. Patients with post-TBI psychosis have shown positive responses to a variety of medications, such as antipsychotics, anticonvulsants, mood stabilizers, and antidepressants [14]. Current evidence, although limited to case reports and case series, suggests that atypical antipsychotics be given as first-line [16]. Among the atypical agents, clozapine seems to be the most potent and effective. However, as it is associated with several and severe side effects, such as agranulocytosis, orthostatic hypotension, drooling, sedation, weight gain, and epileptic seizures, it is not commonly the first to try in a patient with brain injury. It seems rational to try at least two other atypical agents before starting a clozapine trial. A low-dose risperidone (0.5 mg), olanzapine (2.5 mg), or quetiapine (50 mg) can be initiated and then slowly increased, while monitoring for any benefits and adverse events [16]. In our case, the patient has already been started on quetiapine and paliperidone, the primary active metabolite of risperidone, exerting comparable effects [17]. Keeping in mind that avoiding polypharmacy is beneficial, and making one medication adjustment at a time can encourage patient adherence and prevent confusion as to the cause of subsequent clinical outcomes, we decided to optimize her paliperidone, which seems beneficial for psychosis following TBI according to a case report [18].

As management of brain disorders is transforming, it is indeed “time for brain medicine” [19]. Given their potential overlaps in diagnostic and treatment armamentaria, different “brain specialties” can come together and share knowledge and skills following the innovative BMC model to arrive at a unified approach to patient care while creating efficiencies for different stakeholders, for example, patients, families, and healthcare system [5, 19]. The BMC offers concurrent access to more than two brain-related disciplines and provides comprehensive management to distraught patients. Such an innovative model housing different expertise can potentially enhance access to specialized care, save patient and clinician time, conserve healthcare resources, and convey a consistent and consolidated health-related message to patients. Furthermore, since the BMC also involves a fellowship program, wherein trainees from different disciplines pick up skills outside their original training, future generations of “brain specialists” are being molded to handle complex cases through a comprehensive brain–mind–body approach [5].

In our case, the BMC was able to finally provide diagnostic clarity for an elderly woman who had been suffering from neuropsychiatric symptoms over the past 2.5 years. After developing a thorough neurobiopsychosocial formulation from an interdisciplinary lens, we recommended an individualized holistic treatment for the patient. Post-TBI psychosis can benefit from various interventions, ranging from a thorough patient and family education and medication review to optimization of psychotropic medications and appropriate rehabilitation interventions, for example, behavioral, cognitive, functional, vocational, and social skills training [16].

## 4. Conclusion

Schizophrenia-like psychosis after TBI is rare and functionally impairing. Psychosis can occur after mild TBI with neuroimaging evidence of structural intracranial injury (previously known as “complicated” mild TBI). This case report of an elderly patient who presented with post-TBI psychosis exemplifies the utility of a brain medicine model, an expanded shared care model for the diagnosis and management of complex brain disorders.

## Data Availability

The data that support the findings of this report are available within the manuscript.

## References

[1] James S. L., Theadom A., Ellenbogen R. G. (2019). Global, Regional, and National Burden of Traumatic Brain Injury and Spinal Cord Injury, 1990–2016: A Systematic Analysis for the Global Burden of Disease Study 2016. *The Lancet Neurology*.

[2] Fujii D., Ahmed I. (2002). Characteristics of Psychotic Disorder Due to Traumatic Brain Injury. *The Journal of Neuropsychiatry and Clinical Neurosciences*.

[3] Fujii D. E., Ahmed I. (2014). Psychotic Disorder Caused by Traumatic Brain Injury. *Psychiatric Clinics of North America*.

[4] Fujii D. E., Ahmed I. (2001). Risk Factors in Psychosis Secondary to Traumatic Brain Injury. *The Journal of Neuropsychiatry and Clinical Neurosciences*.

[5] Levitt S., Henri-Bhargava A., Hogan D. B., Shulman K., Mitchell S. B. (2023). The Brain Medicine Fellowship: A Competency-Based Training Program to Treat Complex Brain Disorders. *Academic Medicine*.

[6] Broemeling A.-M., Watson D., Prebtani F. (2008). Population Patterns of Chronic Health Conditions, Co-Morbidity and Healthcare Use in Canada: Implications for Policy and Practice. *Healthcare Quarterly*.

[7] Teasdale G., Maas A., Lecky F., Manley G., Stocchetti N., Murray G. (2014). The Glasgow Coma Scale at 40 years: Standing the Test of Time. *The Lancet Neurology*.

[8] Nasreddine Z. S., Phillips N. A., Bédirian V. (2005). The Montreal Cognitive Assessment, MoCA: A Brief Screening Tool For Mild Cognitive Impairment. *Journal of the American Geriatrics Society*.

[9] Darvesh S., Leach L., Black S. E., Kaplan E., Freedman M. (2005). The Behavioural Neurology Assessment. *Canadian Journal of Neurological Sciences/Journal Canadien des Sciences Neurologiques*.

[10] Gurin L., Arciniegas D. B. (2019). Psychosis After Traumatic Brain Injury: Conceptual and Clinical Considerations. https://www.psychiatrictimes.com/view/psychosis-after-traumatic-brain-injury-conceptual-and-clinical-considerations.

[11] Schwarzbold M., Diaz A., Martins E. T. (2008). Psychiatric Disorders and Traumatic Brain Injury. *Neuropsychiatric Disease and Treatment*.

[12] Joseph B., Narayanaswamy J. C., Venkatasubramanian G. (2015). Insight in Schizophrenia: Relationship to Positive, Negative and Neurocognitive Dimensions. *Indian Journal of Psychological Medicine*.

[13] Tagliaferri F., Compagnone C., Korsic M., Servadei F., Kraus J. (2006). A Systematic Review of Brain Injury Epidemiology in Europe. *Acta Neurochirurgica*.

[14] Mcgee J., Alekseeva N. (2016). Traumatic Brain Injury and Behavior: A Practical Approach. *Neurologic Clinics*.

[15] Silverberg N., Iverson G., Cogan A. (2023). The American Congress of Rehabilitation Medicine Diagnostic Criteria for Mild Traumatic Brain Injury. *Archives of Physical Medicine and Rehabilitation*.

[16] McAllister T. W., Ferrell R. B., McAllister T. W. (2002). Evaluation and Treatment of Psychosis after Traumatic Brain Injury. *NeuroRehabilitation [Internet]*.

[17] National Institute of Diabetes and Digestive and Kidney Diseases (2012). LiverTox: Clinical and Research Information on Drug-Induced Liver Injury. https://www.ncbi.nlm.nih.gov/books/NBK548506/.

[18] Douglass A. R., Smyth U. (2018). A Case Report of Guardian-Consent Forced Paliperidone Palmitate for Behavioral Disturbance Due to Traumatic Brain Injury. *Mental Health Clinician*.

[19] Brown J. C., Dainton-Howard H., Woodward J. Time for Brain Medicine. *The Journal of Neuropsychiatry and Clinical Neurosciences*.

